# Higher exercise tolerance early after allogeneic hematopoietic stem cell transplantation is the predictive marker for higher probability of later social reintegration

**DOI:** 10.1038/s41598-021-86744-8

**Published:** 2021-03-30

**Authors:** Ryota Hamada, Yasuyuki Arai, Tadakazu Kondo, Kazuhiro Harada, Masanobu Murao, Junsuke Miyasaka, Michiko Yoshida, Honami Yonezawa, Manabu Nankaku, Sayako Ouchi, Wakako Kitakubo, Tomoko Wadayama, Junya Kanda, Akifumi Takaori-Kondo, Ryosuke Ikeguchi, Shuichi Matsuda

**Affiliations:** 1 Rehabilitation Unit, Kyoto University Hospital, Kyoto, Japan; 2 Department of Physical Therapy, Graduate School of Health Science, Kibi International University, Okayama, Japan; 3 Department of Hematology and Oncology, Graduate School of Medicine, Kyoto University, Kyoto, Japan; 4 Department of Clinical Laboratory Medicine, Graduate School of Medicine, Kyoto University, Kyoto, Japan; 5 Nursing Department, Kyoto University Hospital, Kyoto, Japan

**Keywords:** Haematopoietic stem cells, Rehabilitation

## Abstract

As the proportion of long-term survivors after allogeneic hematopoietic stem cell transplantation (allo-HSCT) is on the rise, it is essential to consider the significance of quality of life (QOL), including reintegration with society (returning to school or work). This retrospective cohort study aims to illustrate the precise epidemiology of social reintegration later after allo-HSCT and determine its predictive indicators. We enrolled 56 patients, and 40 patients (71%) attained social reintegration at 2 years post-HSCT. Reintegration failure markedly correlated with an inferior performance status and concurrent chronic graft-versus-host disease. In non-reintegrated patients, the physical function at discharge measured by the 6-min walking distance (6MWD) was markedly decreased. On the multivariate risk analyses, sex (female; odds ratio (OR) 0.07; 95% confidence interval (CI) 0.01–0.54; *p* = 0.01), HCT-CI (≥ 2; OR 0.10; 95% CI 0.01–0.84; *p* = 0.03), and change in 6MWD (per 5% increase; OR 1.47; 95% CI 1.01–2.13; *p* = 0.04) were significant predictors of later social reintegration. This study suggests that a multidisciplinary strategy including rehabilitation is essential, especially in patients with poor predictive markers at an early phase, and we should consider suitable rehabilitation programs to prevent a decline in exercise tolerance and improve social reintegration and overall QOL in patients after allo-HSCT.

## Introduction

Allogeneic hematopoietic stem cell transplantation (allo-HSCT) is a curative treatment for hematological malignancies. Recently, there have been a remarkable increase in the 5-year survival rate post-HSCT because of the sophisticated conditioning regimens and the development of supportive care for post-transplant complications^1^. With the increasing proportion of long-term survivors after allo-HSCT, quality of life (QOL) could be equally significant. In particular, reintegration with society (returning to school or work) after allo-HSCT is a major factor to enhance the QOL in socially active younger patients^2–5^.

However, reintegration in society is not easily attained after allo-HSCT. Reportedly, a median of 2 years is necessary for patients to return to work^6^, and unemployment remains exorbitant in this population. As the precise data on social reintegration remain limited, the crucial parameters for patients at the timing of their reintegration also remain unclear. In addition, correlation analyses focusing on social reintegration and patients’ factors at pre- and early post-HSCT have been performed only in one pediatric study; later unemployment rates were persistently high in adult patients who survived the previous childhood allo-HSCT, especially in those with a younger age at HSCT, lower performance status (PS) before HSCT, related bone marrow grafts, and myeloablative conditioning regimens with total body irradiation^7^. However, no studies dealing with adult reintegration, have been published, and there are no ways to predict a patient’s future probability of reintegration at the early phase after allo-HSCT. Thus, such data and analyses help to establish effective follow-up systems for long-term survivors with multidisciplinary care teams comprising physicians, nurses, therapists, and other professional healthcare providers^8^.

A previous study indicated that unemployment and following social non-reintegration could be related to the concurrent declined physical function at 1-year post-HSCT^9^. In addition, our study and others have reported that the physical function, including muscle strength and exercise tolerance, often decrease in the early phase post-transplantation because of post-transplant complications such as acute graft-versus-host disease (aGVHD) and declined physical activity^10–14^. Besides, it takes as long as 1 year to restore such declined physical function^6^. Overall, the studies mentioned above suggest that the physical function (muscle strength and exercise tolerance) in early post-HSCT could be a risk factor predicting the later prolonged decline in the physical function and the following lower probability of social reintegration.

Hence, this study aims to (1) illustrate the precise epidemiology of social reintegration 2 years after allo-HSCT and related patients’ factors, and (2) explore the correlation of the social reintegration of long-term survivors with early post-HSCT physical function, and other transplant-related factors. This study could be beneficial to plan more effective and patient-oriented multidisciplinary strategies, including rehabilitation, which could lead more patients to social reintegration and a superior QOL during the lengthy clinical courses after allo-HSCT.

## Subjects and methods

### Eligibility criteria

From the database, we obtained data on adult patients (age: 18–60 years) who underwent allo-HSCT at Kyoto University Hospital (Kyoto, Japan) between April 2011 and April 2018^15–17^. We excluded patients if early death or relapse (before 2 years after allo-HSCT) was recorded, were unemployed before HSCT (had no full-time or part-time job), or had not attended school before HSCT. In this study, “before HSCT” indicates the time of diagnosis for hematological disease. This study adhered to the tenets of the Declaration of Helsinki by the World Medical Association and the ethical guidelines for medical research involving humans and was approved by the Institutional Review Board of Kyoto University (approval number: R0715). We observed an informed consent from all the participants involved in the study.

### Data collection and definition of variables

We obtained descriptive data from the database and included the following variables: sex, age, body mass index (BMI), ECOG (Eastern Cooperative Oncology Group) PS (Performance Status) as a commonly used performance status score, hematopoietic cell transplantation comorbidity index (HCT-CI), diagnosis, stem cell source, disease status, conditioning, aGVHD, time to neutrophil engraftment, hospitalized period after HSCT, and evaluation results at discharge (BMI, hemoglobin, serum total protein, serum albumin, and C-reactive protein levels). Hemoglobin was used as the marker related to exercise tolerance^18^, while BMI, total protein, and albumin as indicators for nutrition, and C-reactive protein as inflammatory marker. Nutrition and inflammation were closely related to exercise tolerance^19^.

In this study, social reintegration was defined as returning to paid work or school and evaluated at 2 years after allo-HSCT. Of note, patients working in non-paid style, such as volunteer and housework, were categorized as being socially non-reintegrated. Work pattern was defined by working hours, and full-time work was defined as 40 h or more per week, while part-time work as < 40 h per week. Furthermore, the social reintegration status was established by specialized nurses in an outpatient clinic during long-term follow-up sessions.

### Protocols for rehabilitation and assessments of physical function

All enrolled patients used the rehabilitation protocol. The study cohort was under rehabilitation interventions during hospitalized periods between pre-HSCT (before starting conditioning regimens) and discharge^11^. For the patients before engraftment, the rehabilitation program was performed in clean rooms, where the interventions mainly focused on maintaining personal activities and physical functions. The exercise therapies were performed every weekday (five times a week) for 20–40 min, and involved stretching, strength training, walking, and bicycle ergometer. The exercise load was set at “somewhat strong” according to the revised Borg scale, and the target heart rate was set using Karvonen method with exercise loads of 40%–60%^20^. Notably, rehabilitation was continued inside the ward even during the aseptic management periods (neutrophil counts < 500/μL)^11^. Even when bed-ridden, the patients were provided with range of motion exercises (ROM practice) and muscle training to prevent contractures and muscle weakness on the bed.

In the patients after engraftment, the rehabilitation program was performed in the rehabilitation center, where the interventions mainly focused on improving the physical function and re-acquiring activities of daily living (ADL) movement for discharge. Activities included ADL training (stair climbing practice and floor movement practice) and the training in clean rooms. The intervention time could attain 1 h, and the exercises load was set at “somewhat strong” or rather higher “strong” levels.

Besides rehabilitation interventions, the physical function was routinely assessed using standardized tests of the knee extensor strength (KES) for muscle strength^21^ and the 6-min walking distance (6MWD) for exercise tolerance^11^. In this study, the KES was measured during a 3-s isometric contraction using IsoForce GT-330 (OG Giken Co., Ltd., Okayama, Japan). Precisely, patients were seated on a chair keeping the angles of their hip and knee joints at 90° and instructed to extend the knee joints (to kick forward); then, the force was measured with a sensor placed 5 cm above the lateral malleolus. We used the higher score (N) obtained from two trials in future analyses. In addition, the torque was calculated by multiplying strength by the lever arm length and was expressed as the ratio to body weight (Nm/kg)^21^. Besides, the 6MWD test was conducted as recommended by the American Thoracic Society^22^, where patients were requested to walk back and forth along a straight 30-m course on the corridor at the maximum efforts for 6 min, and the walking distance was recorded. The reported 6MWD in the healthy control ranged from 494 to 631 m according to the sex, age, height, and weight ^23,24^. The physical function tests (KES and 6MWD) were assessed pre-transplantation (before conditioning regimens) and just before discharge (1 week before discharge).

### Statistical analysis

The statistical analyses were performed using SPSS software ver. 18.0 (IBM SPSS, Inc., Armonk, NY). A two-sided *p* < 0.05 was considered statistically significant. Using the Mann–Whitney *U*-test and unpaired *t*-test, we compared the patients’ characteristics, including the physical function between socially reintegrated and non-reintegrated groups. In addition, we evaluated the magnitude of change in the physical function (Δ) at discharge compared with at pre-HSCT. Furthermore, we used the paired *t*-test to compare changes in the physical function within each group over time.

In addition, we used the univariate simple logistic regression test to calculate the unadjusted odds ratio (OR), 95% confidence intervals (CI), and *p* value to determine the correlation between pre- or post-HSCT characteristics and later social reintegration. Moreover, we performed multivariate logistic regression analyses with dependent variables using variables with a *p* < 0.2 in univariate analyses. We used the forced entry method to build the model. In the power analyses where the two subgroups are compared, and the social reintegration incidence differed in three times, total 42 patients were necessary to detect the significant difference with an alpha error of 0.05.

## Results

### Patients’ characteristics

**Figure 1:**
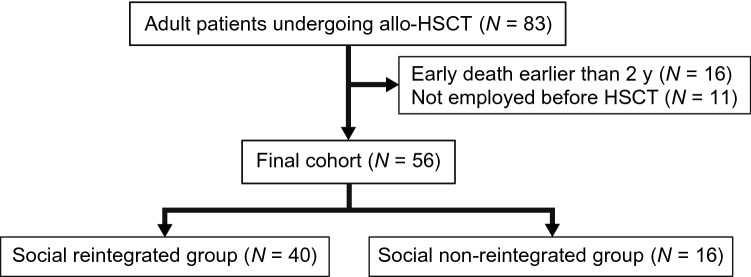
The study flow diagram. The flow diagram of the study cohort is shown. The inclusion and exclusion criteria were compatible with those shown in Methods.

During the study period, 83 patients underwent allo-HSCT. Of these, we excluded 27 patients because of early death or relapse (*N* = 16) and non-working/studying status pre-HSCT (*N* = 11). We finally enrolled 56 patients (Fig. 1). All the 11 patients excluded because they were not working/studying before HSCT were still unemployed after transplantation. Table 1 presents the patients’ characteristics. Overall, 75% of the study cohort were male patients (because we excluded unemployed patients at HSCT), and the median age at HSCT was 43.5 years. In addition, the ECOG PS was relatively fair (PS 0–1 in 96%). The HCT-CI was 0–1 in 80% of the cohort, and patients with high scores (*N* = 4, HCT-CI 3) had respiratory dysfunction with arrythmia or diabetes, or rheumatoid disease with inflammatory colitis. Regarding the disease status, 75% of the cohort was transplanted at complete remission (because we excluded patients with relapse or early death within 2 years post-HSCT).

**Table 1:** Patient background, treatment and physical function during hospitalization.

Variables		Total *N* = 56 (%)	Social reintegration		*p*
			Y (*N* = 40)	N (*N* = 16)	
**Pre-HSCT characteristics**					
Sex	Male/Female	42 (75%)/14(25%)	33 (83%)/7(17%)	9 (56%)/7(44%)	0.04*
Age, y	Median (range)	43.5 (17–60)	43.0 (17–60)	47.5 (31–60)	0.43
BMI, kg/m^2^	Mean (range)	22.0 (16.0–33.2)	21.9 (16.0–33.2)	22.2 (16.1–31.1)	0.68
PS	0–1/2–4	54 (96%)/2(4%)	38 (95%)/2(5%)	16 (100%)/0(0%)	0.67
HCT-CI	0–1/2-	45 (80%)/11(20%)	35 (88%)/5(12%)	10 (63%)/6(37%)	0.04*
Diagnosis	AML	19 (34%)	15 (38%)	4 (25%)	0.81
	MDS	9 (16%)	6 (15%)	3 (19%)	
	ALL	17 (30%)	12 (30%)	5 (31%)	
	ML	11 (20%)	7 (17%)	4 (25%)	
Stem cell source	Rel-BM	6 (10%)	4 (10%)	2 (12%)	0.55
	Rel-PB	21 (38%)	13 (32%)	8 (50%)	
	UR-BM	7 (13%)	6 (15%)	1 (6%)	
	CB	22 (39%)	17 (43%)	5 (32%)	
Disease status	CR/nCR	42 (75%)/14(25%)	31 (78%)/9(22%)	11 (69%)/5(31%)	0.49
Conditioning	MAC/RIC	39 (70%)/17(30%)	27 (68%)/13(32%)	12 (75%)/4(25%)	0.58
**Post-HSCT characteristics**					
aGVHD	all/Gr2-4	30 (54%)/22(40%)	23 (58%)/16(40%)	7 (43%)/6(38%)	0.86
Neut engraft, d	Median (range)	21.0 (11–140)	19.5 (11–57)	25.0 (19–140)	0.62
**Variables at discharge**					
Hospitalized period, d	Median (range)	69.0 (32–149)	64.0 (32–149)	72.0 (33–126)	0.34
BMI, kg/m^2^	Mean (range)	20.1 (15.4–30.7)	20.1 (16.0–30.7)	19.9 (15.4–26.5)	0.55
PS	0–1/2–4	52 (93%)/4(7%)	39 (98%)/1(2%)	13 (81%)/3(19%)	0.06
Hgb, g/dL	Mean (range)	9.4 (6.8–14.7)	9.6 (6.8–14.7)	8.8 (7.0–11.6)	0.10
TP, g/dL	Mean (range)	5.8 (4.3–7.2)	6.5 (4.3–7.2)	6.6 (4.5–6.6)	0.56
Alb, g/dL	Mean (range)	3.6 (2.6–4.8)	3.7 (2.6–4.8)	3.5 (3.1–3.9)	0.40
CRP, mg/dl	Mean (range)	0.5 (0.0–4.3)	0.3 (0.0–4.3)	0.1 (0.0–0.3)	0.86

Y; yes, N; no, HSCT; hematopoietic stem cell transplantation, BMI; body mass index, PS; performance status, HCT-CI; hematopoietic cell transplantation comorbidity index, AML; acute myeloid leukemia, MDS; myelodysplastic syndrome, ALL; acute lymphoblastic leukemia, ML; malignant lymphoma, Rel; related, UR; unrelated, BM; bone marrow, PB; peripheral blood, CB; cord blood, CR; complete remission, nCR; nonCR, MAC; myeloablative conditioning, RIC; reduced intensity conditioning, aGVHD; acute graft-versus-host disease, Gr; grade, Neut; neutrophil, Hgb; hemoglobin, TP; serum total protein, Alb; serum albumin, and CRP; C-reactive protein. *indicates statistically significant.

### Social reintegration after allo-HSCT

**Figure 2:**
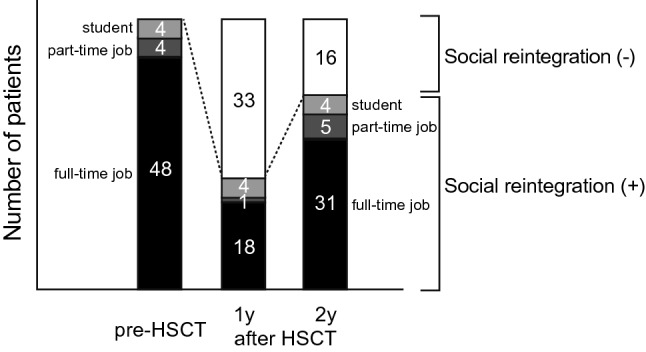
Social integration status pre- and post- HSCT. Social status pre-HSCT and at 1 year and 2 years post-HSCT are shown. The number denotes the number of patients in each category.

From 23 patients (41%) at 1-year post-HSCT, social reintegration increased to 40 patients (71%) at 2 years post-HSCT. In the socially reintegrated group, 35 patients returned to full-time work or school, while five patients acquired a part-time job (Fig. 2 and Supplemental Table 1). Among social non-integrated patients at 2 years (*N* = 16), one patient was categorized as a house worker, while the other patients were not employed either in the paid or non-paid style. Regarding social reintegration, in both groups, the patient distributions were almost similar in age, BMI, disease type or status, stem cell sources, and other post-HSCT variables, whereas the distribution of sex and HCT-CI was skewed (Table 1).

### Physical function evaluation

**Figure 3:**
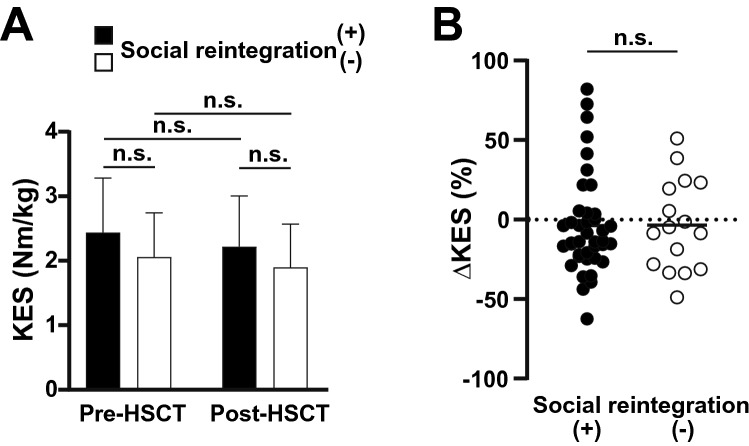
The change of the knee extensor strength (KES) and the correlation with social reintegration. (**A**) Measured KES values (mean ± standard deviation) are shown based on the later social reintegration status at each time-point of pre- and post-HSCT. (**B**) KES changes (ΔKES) are plotted individually. n.s., not significant.

In all patients, rehabilitation was performed during the hospital stay, and the median adherence was 83% (range, 43–100); adherence was not related to the future social reintegration (OR 1.92, 95% CI 0.59–6.23, *p* = 0.27). The physical function was assessed by the KES (as markers for muscle strength) and the 6MWD (as markers for exercise tolerance) at two time-points of pre-HSCT and at discharge. The KES was equivalent in pre- and post-HSCT periods (mean ± standard deviation; before: 2.34 ± 0.82 Nm/kg *vs.* after: 2.15 ± 0.77 Nm/kg; *p* = 0.08); this tendency was similar in each group of future social reintegration (Fig. 3A, comparison of first *vs.* third columns, and second *vs.* fourth columns). Besides, we observed no significant difference between both groups at each time-point (Fig. 3A, comparison of first *vs.* second columns, and third *vs.* fourth columns). Furthermore, ΔKES (difference between pre- and post-HSCT) did not significantly differ between both groups (socially reintegrated *vs.* non-reintegrated group; mean, − 3.7% *vs.* − 3.3%; *p* = 0.96; Fig. 3B). The absolute KES values in each patient are displayed in Supplemental Fig. 1A.

**Figure 4:**
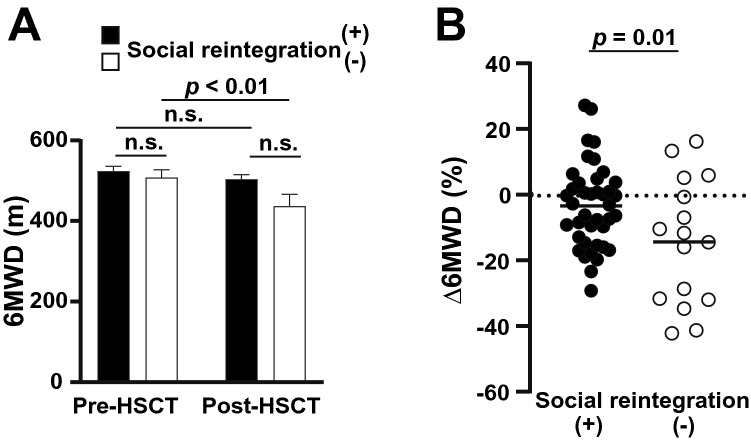
The change of the 6-min walking distance (6MWD) and the correlation with social reintegration. (**A**) Measured 6MWD values (mean ± standard deviation) are shown based on the later social reintegration status at each time-point of pre- and post-HSCT. (**B**) 6MWD changes (Δ6MWD) are plotted individually.

The 6MWD exhibited more significant and specific changes. Overall, the 6MWD significantly declined in post-HSCT (522.0 ± 76.4 m *vs.* 486.9 ± 93.3 m; *p* = 0.01); this was not observed in the future social reintegration group (before: 526.6 ± 76.9 m, *vs.* after: 506.0 ± 74.4 m; *p* = 0.13; Fig. 4A, comparison of first *vs.* third columns). In the non-reintegration group, however, the difference between these two time-points was apparent and statistically significant (before: 510.5 ± 76.4 m, *vs.* after: 439.2 ± 118.0 m; *p* < 0.01; Fig. 4A, comparison of second *vs.* fourth columns). The 6MWD decline in the future non-social reintegration group was more apparent in the analyses for Δ6MWD (mean, − 3.0% in the integration group *vs.* − 14.2% in the non-integration group; *p* = 0.01; Fig. 4B). The absolute 6MWD values in each patient are displayed in Supplemental Fig. 1B. In addition, Δ6MWD was significantly related to the high HCT-CI score (HCT-CI ≥ 2, *p* < 0.01).

### Patients’ clinical status at 2 years post-HSCT and social reintegration

Next, we examined the patients’ clinical status at 2 years post-HSCT to determine the factors directly related to the patient’s social reintegration. Among various factors, including BMI, PS, chronic GVHD (cGVHD), hemoglobin, total protein, serum albumin, and C-reactive protein, the proportion of patients with inferior PS and cGVHD was significantly skewed in the non-reintegrated group (*p* < 0.01; Table 2), suggesting that these two factors could directly affect social reintegration.

**Table 2:** Patient status at 2 years after allo-HSCT.

Variables		Total *N* = 56 (%)	Social reintegration		*p*
			Y (*N* = 40)	N (*N* = 16)	
BMI, kg/m^2^	Mean (range)	21.7 (16.7–31.6)	21.6 (16.7–31.6)	21.6 (18.1–26.8)	0.97
PS	0–1/2–4	48 (86%)/8 (14%)	40 (100%)/0 (0%)	8 (50%)/8 (50%)	< 0.01*
cGVHD	Y	28 (50%)	16 (40%)	12 (75%)	< 0.01*
Hgb, g/dL	Mean (range)	12.7 (6.9–17.5)	12.7 (10.4–17.5)	11.5 (6.9–15.4)	0.09
TP, g/dL	Mean (range)	6.6 (5.3–7.6)	6.6 (5.6–7.6)	6.4 (5.3–7.5)	0.34
Alb, g/dL	Mean (range)	4.1 (3.1–5.1)	4.1 (3.6–5.1)	3.9 (3.1–4.6)	0.12
CRP, mg/dl	Mean (range)	0.3 (0.0–0.6)	0.3 (0.0–0.6)	0.1 (0.0–0.3)	0.15

BMI; body mass index, PS; performance status, cGVHD; chronic graft-versus-host disease, Y; yes, Hgb; hemoglobin, TP; serum total protein, Alb; serum albumin, and CRP; C-reactive protein. *indicates statistically significant.

### Factors related to future social reintegration

In order to identify the factors that can predict social reintegration in the later phase post-HSCT, we sought to establish the logistic regression model using pre-HSCT patient characteristics and those with early post-HSCT. The univariate analyses revealed that the patients’ sex, HCT-CI, PS, hemoglobin, albumin at discharge, and 6MWD were relevant to the incidence of social reintegration (Table 3). The median cumulative dosage of corticosteroids was 3.2 mg/kg (range, 0–91.5), and we found no clear relationship with social reintegration (OR 0.70; 95%CI 0.21–2.55; *p* = 0.55). Owing to the multivariate analyses using variables extracted in the univariate analyses, sex (female; OR 0.07; 95% CI: 0.01–0.54; *p* = 0.01), higher HCT-CI (OR 0.10; 95% CI 0.01–0.84; *p* = 0.03), and Δ6MWD (per 5% increase; OR 1.47; 95% CI: 1.01–2.15; *p* = 0.04) were identified as significant risk factors that predict the incidence of social reintegration (Table 3). Notably, increase in 6MWD significantly correlated with the higher incidence of social reintegration. Other factors, including ΔKES, were statistically nonsignificant, and the correlation with reintegration was not apparent. The pre-HSCT employment pattern (full- *vs.* part-time) had no significant impact on the probability of post-HSCT reintegration.

**Table 3:** Uni-and multi-variate analyses of patients’ factors affecting social reintegration.

Variables		Univariate		Multivariate	
		OR (95%CI)	*p*	OR (95%CI)	*p*
**Pre- and post-HSCT characteristics**					
Sex	Female	0.27 (0.76–0.98)	0.04*	0.07 (0.01–0.54)	0.01*
Age	40 y or over	0.61 (0.18–2.10)	0.43		
BMI	< mean	0.75 (0.19–2.95)	0.68		
PS	2–4	0.11 (0.01–1.16)	0.67		
HCT-CI	2-	0.23 (0.60–0.94)	0.04*	0.10 (0.01–0.84)	0.03*
Diagnosis	AML	1.00 (reference)			
	MDS	0.76 (0.16–3.51)	0.73		
	ALL	0.94 (0.26–3.30)	0.92		
	ML	0.63 (0.15–2.56)	0.52		
Stem cell source	Rel-BM	1.00 (reference)			
	Rel-PB	2.64 (0.29–23.9)	0.38		
	UR-BM	0.48 (0.14–1.57)	0.22		
	CB	1.62 (0.47–5.55)	0.43		
Disease status	nCR	0.63 (0.17–2.32)	0.49		
Conditioning	MAC	0.69 (0.18–2.56)	0.58		
aGVHD	Gr2-4	1.11 (0.33–3.66)	0.86		
**Variables at discharge**					
Hospitalized period	> median	0.65 (0.26–1.60)	0.34		
BMI	< mean	1.42 (0.44–4.56)	0.55		
PS	2–4	0.42 (0.16–1.06)	0.06	0.65 (0.02–19.3)	0.80
Hgb	< mean	0.37 (0.10–1.28)	0.10	0.27 (0.04–1.83)	0.18
TP	< mean	0.56 (0.16–1.91)	0.35		
Alb	< mean	0.40 (0.12–1.36)	0.14	0.57 (0.11–3.03)	0.51
CRP	< mean	0.86 (0.26–2.76)	0.79		
**Evaluation for physical function at discharge**					
KES (per 0.5Nm/kg change)		1.33 (0.87–2.01)	0.17		
∆KES (per 5% change)		1.00 (0.90–1.09)	0.96	0.94 (0.80–1.10)	0.46
6MWD (per 5 m change)		1.04 (1.00–1.08)	0.02*		
∆6MWD (per 5% change)		1.28 (1.03–1.58)	0.02*	1.47 (1.01–2.15)	0.04*

OR; odds ratio, CI; confidence interval, HSCT; hematopoietic stem cell transplantation, BMI; body mass index, PS; performance status, HCT-CI; hematopoietic cell transplantation comorbidity index, AML; acute myeloid leukemia, MDS; myelodysplastic syndrome, ALL; acute lymphoblastic leukemia, ML; malignant lymphoma, Rel; related, UR; unrelated, BM; bone marrow, PB; peripheral blood, CB; cord blood, nCR; non complete remission, MAC; myeloablative conditioning, aGVHD; acute graft-versus-host disease, BMI; body mass index, PS; performance status, Hgb; hemoglobin, TP; serum total protein, Alb; serum albumin, and CRP; C-reactive protein, KES; knee extensor strength, and 6MWD; 6-min walking distance. ∆ indicates difference between pre- and post-HSCT. * indicates statistically significant.

Subgroup analyses on the age (≤ 45 *vs.* > 45 years) indicated that our main results (high HCT-CI and large decline in 6MWD as risk factors for social non-reintegration) can be reproducible in each age group (data not shown).

## Discussion

This single-centered retrospective cohort study exploring social reintegration in post-allo-HSCT patients (under rehabilitation interventions during hospitalized periods between pre-HSCT and discharge) revealed these two major findings: (1) social reintegration was achieved in 71% of patients with long-term survival, and the concurrent poor PS (≥ 2) and cGVHD exerted a significant impact on the inferior reintegration; and (2) the 6MWD changed early after allo-HSCT (at discharge), including the patient sex and HCT-CI, could predict the probability of the social reintegration status at 2 years post-HSCT. To the best of our knowledge, this is the first study to reveal the correlation between social reintegration and early physical function, and suggest the significance of rehabilitation intervention to attain a higher incidence of social reintegration and a better QOL after allo-HSCT^5^.

First, this study illustrated the accurate status of social reintegration 2 years after allo-HSCT through the face-to-face survey at the outpatient unit. Among various parameters, employment and returning to school are the most important markers of functional recovery in cancer survivors^25,26^. Specifically, employment after HSCT is an indicator of post-transplantation recovery and function with economic and social implications^9^. In accordance with our study (71% at 2 years), other studies reported an 81%–89% of social reintegration at 5 year post-transplant^27,28^. In this study, we focused on reintegration not at 5 but 2 years post-HSCT, primarily because this is the earliest timing when the risk of infection and cGVHD is saturated^29^, and also because previous studies have indicated that 1–2 years is the median time needed for patients to return to their work^6,30^.

Even at 2 years after allo-HSCT, as many as 29% of our cohort were socially not-reintegrated; thus, we focused on the factors associated with social reintegration. A previous study reported that HSCT complication-associated deterioration of PS correlated with health-related failure to return to work or school^31^. In this study, poor PS concurrent cGVHD, at 2 years markedly correlated with the lower incidence of social reintegration. Notably, cGVHD encompasses various symptoms, including the eyes, mouth, skin, nail, hair, gastrointestinal tract, lungs, liver, muscles and joints, and genitalia^29^, together with additional immunosuppressive therapies could deteriorate the patients’ PS and warrant frequent hospital visits or admissions; these situations could hinder patients from reemployment or returning to school^32^.

Second, we performed the analyses to identify the variables (pre- or early post-HSCT) that could predict later social reintegration. The multivariate analyses suggested that female sex, higher HCT-CI, and changes in the 6MWD at discharge were significant factors. Regarding sex, our findings corroborated previous studies^27,28^. It is reported that gender wage gap in Japan is the third largest in the OECD (Organization for Economic Co-operation and Development) area^33^, and female HSCT recipients may be less liable to return to work, which can explain the skewed distribution of the included patients’ sex and the post-HSCT lower incidence of reintegrated females. On the other hand, this is the first study to report the correlation between higher HCT-CI and lower reintegration. Patients with high HCT-CI scores are complicated with dysfunctions in single or multiple organs per se or because of chemo/radiotherapies pre-HSCT^34^. Perhaps, such pre-transplant complications could be frequently carried over or deteriorate during the clinical courses after allo-HSCT^16^, resulting in lower PS and poorer social reintegration, as discussed above.

Besides the female sex and higher HCT-CI, correlation with the declined 6MWD and a lower incidence of social reintegration is a novel non-elucidated finding. The 6MWD test is a standardized assessment of exercise tolerance and is often used in allo-HSCT patients. Although some studies have reported a decreased distance early post-HSCT^35,36^, this study is the first to compare patients with and without social reintegration. In addition, the superiority of the 6MWD at discharge was established in the socially integrated group, although their hospital stay was shorter (the 6MWD measurement at an earlier time-point post-HSCT in this group working as a bias), supporting the earlier recovery or improvement of 6MWD in the future reintegrated group. We found that changes can be a predictive factor for the future social reintegration; this suggests that recovery or improvement is the key for better reintegration. On the other hand, the KES test, which indicates instantaneous muscle strength^21^, exhibited a nonsignificant difference between both groups.

These findings suggested that among various physical functions, insufficient exercise tolerance (6MWD) at an early time-point post-HSCT, not the loss of instantaneous muscle strength (KES), could interfere with subsequent social reintegration over time. To be socially reintegrated after allo-HSCT, it is speculated that patients need to attain sufficient exercise tolerance, which could overcome the continuous demands of exercise, including long-distance commutation and long-time work without intervals, and this study indicates that tolerance at this time (2 years post-HSCT) could be closely related to early post-HSCT (at discharge). Thus, a multidisciplinary approach to patients with lower exercise tolerance at the early time-point post-HSCT could be crucial for future reintegration, and rehabilitation interventions to maintain (during hospitalization) or restore (after discharge) the exercise tolerance could increase the future social reintegration probability^37^. In Japan especially, rehabilitation programs in the outpatient clinics are not covered by the insurance, and no rehabilitation is provided after discharge. Therefore, insufficient exercise tolerance (shorter 6MWD) cannot be fully restored without appropriate rehabilitation interventions even long after the discharge; this may be the reason why Δ6MWD at discharge is strongly related to the lower social reintegration two years later. Revision of the rehabilitation program to increase the total exercise load using short, higher intensity interval-based protocols can also be helpful to improve cardiorespiratory fitness and following refinement of exercise tolerance^38^, and continuous rehabilitation programs in the outpatient clinics^39^.

Hence, this study revealed the epidemiology of post-HSCT social reintegration and its predictive factors; however, there exist some limitations. First, we did not evaluate physical function (including 6MWD) and QOL while judging social reintegration (2 years post-HSCT). Thus, changes in these parameters long after HSCT should be analyzed in future research to determine the impacts of physical function on the social reintegration. Second, this study was conducted at a single institute within a limited period, and the wide differences were not considered regarding social support services and attitudes for social reintegration based on each region and country where HSCTs were performed as well as decades, patient sex, and age. Of notes, the hospitalization periods in Japan are usually longer (60–170 days) than those in the other countries, because the treatments of GVHD, infection, CMV reactivation, and so on are often performed during the hospital stay^40^. Third, the number of the patients and observational periods are also limited, and we excluded patients in the non-working/studying status pre-HSCT. Moreover, we did not record the magnitude of physical activity during the rehabilitation programs. We did not include various confounding factors after the discharge until 2 years later. Thus, the findings of this study should be validated in future multicentered prospective trials including other countries.

In conclusion, this study demonstrated that social reintegration was attained in 71% of patients 2 years after allo-HSCT, and poorer PS and concurrent cGVHD correlated with a lower chance of social reintegration (reemployment and return to school). In addition, female sex, higher HCT-CI, and declined exercise tolerance (6MWD) early after allo-HSCT were predictive factors of the later lower social reintegration among long-term survivors; all these are crucial indicators for subsequent reintegration and the need of supportive care. Thus, this study suggests that a multidisciplinary strategy, including rehabilitation, is imperative, especially for patients with poor predictive markers at an early phase, and an appropriate rehabilitation program to prevent a decline in exercise tolerance should be considered to improve social reintegration and overall QOL in patients after allo-HSCT.

## Supplementary Information

Supplementary Information
