# Supplementary material for: Higher exercise tolerance early after allogeneic hematopoietic stem cell transplantation is the predictive marker for higher probability of later social reintegration
Source: Sci Rep. 2021 Mar 30;11:7190. doi: 10.1038/s41598-021-86744-8 (PMC8010097; doi:10.1038/s41598-021-86744-8)

Higher exercise tolerance early after allogeneic hematopoietic stem cell transplantation is the predictive marker for higher probability of later social reintegration

Ryota Hamada[1,2], Yasuyuki Arai[3,4], Tadakazu Kondo[3], Kazuhiro Harada[2], Masanobu Murao[1,2], Junsuke Miyasaka[1], Michiko Yoshida[1], Honami Yonezawa[1], Manabu Nankaku[1], Sayako Ouchi[5], Wakako Kitakubo[5], Tomoko Wadayama[5], Junya Kanda[3], Akifumi Takaori-Kondo[3], Ryosuke Ikeguchi[1], Shuichi Matsuda[1]

[1]Rehabilitation Unit, Kyoto University Hospital, Kyoto, Japan, [2]Department of Physical Therapy, Graduate School of Health Science, Kibi International University, Okayama, Japan, [3]Department of Hematology and Oncology, [4]Department of Clinical Laboratory Medicine, Graduate School of Medicine, Kyoto University, Kyoto, Japan, [5]Nursing Department, Kyoto University Hospital, Kyoto, Japan

Supplemental materials

Supplemental Table 1. Change in employment status pre-HSCT *vs.* 2 years after HSCT

Employment status pre-HSCT	Total	Employment status post-HSCT			
		Full-time	Part-time	Student	Not reintegrated
Full-time	48 (100%)	31 (64.5%)	2 (4.2%)	1 (2.1%)	14 (29.2%)
Part-time	4 (100%)	0 (0.0%)	2 (50.0%)	0 (0.0%)	2 (50.0%)
Student	4 (100%)	0 (0.0%)	1 (25.0%)	3 (75.0%)	0 (0.0%)
Total	56 (100%)	31 (55.5%)	5 (8.9%)	4 (7.1%)	16 (28.6%)

Abbreviations: HSCT; hematopoietic stem cell transplantation.

Supplemental Figure Legend

Supplemental Figure 1. Absolute values of KES and 6MWD in each patient

Absolute values of (**A**) KES and (**B**) 6MWD in each patient are plotted according to the HSCT status (pre- *vs.* post-) and future social reintegration status. Each dot indicates the value of one patient.

Supplemental Figure 1

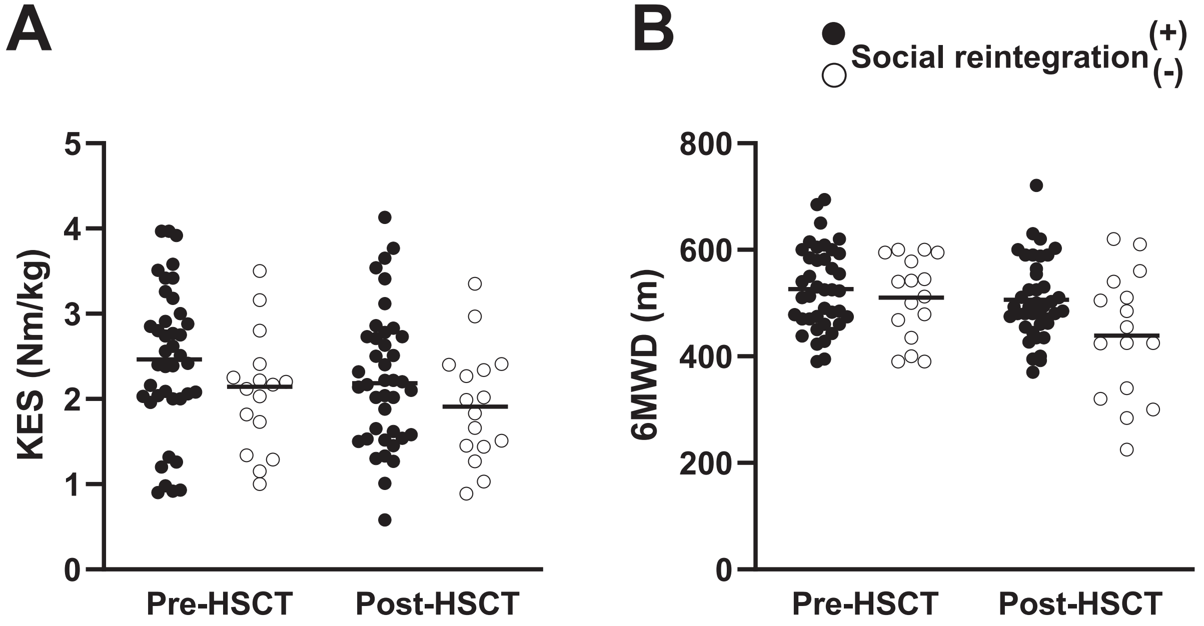

Supplement: Supplementary file 1 — Supplementary Information [file 41598_2021_86744_MOESM1_ESM.pdf]
